# Factors predicting drop out from, and retention in, specialist drug treatment services: A case control study in the North West of England

**DOI:** 10.1186/1471-2458-8-149

**Published:** 2008-05-06

**Authors:** Caryl M Beynon, Alison M McMinn, Adam JE Marr

**Affiliations:** 1 Centre for Public Health, Liverpool John Moores University, Liverpool, L3 2AY, UK; 2 Medical Research Council (MRC) Epidemiology Unit, Cambridge, CB2 0QQ, UK

## Abstract

**Background:**

In the United Kingdom (UK), the National Treatment Agency for Substance Misuse (NTA) considers retention to be the best available measure of drug treatment effectiveness. Accordingly, the NTA has set local treatment systems the annual target of retaining 75% of clients for 12 weeks or more, yet little assessment of this target or factors that improve retention has occurred. This study aims to quantify the proportion of people retained in treatment for 12 weeks in the North West of England and to identify factors associated with premature drop out.

**Methods:**

The North West National Drug Treatment Monitoring System (NDTMS) was used to identify treatment durations for everyone beginning a treatment episode between 1^st ^April 2005 and 31^st ^March 2006 (N = 16626). Odds ratios, chi-square and logistic regression analyses compared clients retained for 12 weeks to clients whose discharge record showed they had prematurely dropped out before 12 weeks. Individuals with other outcomes were excluded from analyses.

**Results:**

75% of clients (N = 12230) were retained for 12 weeks and 10% (N = 1649) dropped out prematurely. Multivariate analysis showed drop out was more likely among Asian drug users (adjusted odds ratio 1.52, 95% CI 1.12 to 2.08) than their white equivalents. Drop out was more likely among residents of Cumbria and Lancashire (adjusted odds ratio 1.80, 95% CI 1.51 to 2.15) and Greater Manchester (adjusted odds ratio 2.00, 95% CI 1.74 to 2.29) than Cheshire and Merseyside and less likely among alcohol users (adjusted odds ratio 0.73, 95% CI 0.59 to 0.91). A significant interaction between age and deprivation was observed. For those aged 18 to 24 years and 25 to 34 years, drop out was significantly more likely among those living in affluent areas. For those in the older age groups the converse effect was observed.

**Conclusion:**

In combination, the drug treatment systems of the North West achieved the Government's retention target in 2005/06. A number of factors associated with drop out were identified; these should be considered in strategies that aim to improve retention. Drop out and retention are measures that capture the joint effect of many factors. Further work is required to evaluate the effect of deprivation.

## Background

According to the World Health Organization, there are an estimated two billion alcohol users, 1.3 billion smokers and 185 million users of illicit drugs in the world. In 2000, these substances contributed to 12.4% of deaths worldwide [1]. While illicit drug use constitutes the smallest proportion of psychoactive substance use, it is the category often perceived as most harmful, and consequently, significant resources are employed globally to prevent and reduce the use of illicit substances. In the United Kingdom (UK), the drugs considered to have the greatest negative consequences are opiates (predominantly heroin) and stimulants (predominantly cocaine and crack cocaine) and it is a reduction in the use of these problematic drugs which continues to be the major focus of the national drug strategy [2].

In many countries, including the UK, effective treatment is understood to play a central role in enabling individuals to adopt drug free lives [2,3]. In recognition of the importance of treatment, the amount allocated by the Department of Health to local partnerships to support drug treatment has swelled to £385 million in 2006/07 [4]. In combination with other measures including an expanded workforce, reduced waiting times and the development of a criminal justice programme designed to divert drug users from crime into treatment, this additional funding saw 195,464 people accessing structured drug treatment between 1^st ^April 2006 and 31^st ^March 2007, compared to 85,000 in 1998 when the national drug strategy was launched [5], a rise of 130%. Increasing treatment uptake is, however, only one measure of success. Unplanned discharge, or 'drop out', is a well-recognised phenomenon of drug treatment, with people dropping out before participating fully in elements of treatment that will engender change [6].

In the United States of America (USA), longer durations of drug treatment are reported to be a consistent predictor of favourable treatment outcomes [7-10] and retaining drug users in treatment should be an important intermediate goal of the treatment system. In the UK, findings from the National Treatment Outcome Research Study, the only major UK-based, prospective cohort treatment outcomes study reported that clients who stayed in residential treatment programmes (programmes which typically see people with more severe problems at intake than those treated in the community) for 'critical times' had better outcomes at one-year follow up than those who were not retained [6]. Drawing on evidence from the USA and UK, the National Treatment Agency for Substance Misuse (NTA), the Government body overseeing the effectiveness of the drug treatment system in the UK, decided that individuals not retained in treatment for at least three months were unlikely to experience long-term benefits [11]. Accordingly, the NTA introduced a retention target in treatment plans for the first time in 2004/05, with 75% of individuals expected to be retained for 12 weeks or more during each annual reporting cycle [12]. Treatment provision, overseen by drug partnerships called Drug (and Alcohol) Action Teams (D [A]ATs), were clearly expected to change their practices in order to achieve this retention target but were obviously not expected to distort their treatment data accordingly. This risk was monitored by the NTA to expose any services and systems that manipulated their delivery in order to appear to achieve this performance indicator, in the absence of any real improvement [11].

To date, there has been little independent assessment of the impact of this government target on the number of drug users actually retained in treatment for 12 weeks. Furthermore, most of the existing evidence regarding factors associated with longer treatment episodes is derived within the USA, with less information available about factors that influence treatment retention in other countries. Factors associated with retention in the UK, where the majority of treatment is delivered via the National Health Service and charitable/voluntary organisations may be very different from those influencing treatment retention in the USA where patients pay for treatment and are discharged for non-payment [13]. In the UK, the factors that influence treatment retention and attrition remain poorly understood.

The North West of England (mid-year population of 6,846,249 in 2005) is a region containing both urban and rural and both deprived and affluent areas. It is also a region with a relatively high prevalence of problematic drug use. While the exact number of drug users living in the region remains unknown due to the covert nature of the behaviour, estimates suggest that the level of problematic drug use is as high as 52 per 1,000 of male population aged 15 to 44 years in some metropolitan centres [14]. Between 1^st ^April 2005 and 31st March 2006, 35503 North West residents were in contact with structured drug treatment services and these individuals accounted for almost 20% of the total number of people in contact with treatment services in England [15]. The North West region was therefore chosen as the setting for this study because it represents an area with a high prevalence of problematic drug use and treatment activity. The study set out to quantify the proportion of individuals retained in treatment in the North West of England for 12 weeks or more and to investigate the factors associated with treatment drop out, compared to retention, using established monitoring systems.

## Methods

### Data collection

The National Drug Treatment Monitoring System (NDTMS) records details of individuals in contact with structured drug treatment services in England. Such services include substitute prescribing, structured psychosocial interventions and abstinence-based services but exclude agencies such as needle exchange services [16]. It is mandatory for staff working in these services to report to their regional NDTMS key items of data relating to all drug users who present to their service for treatment. Data are collected and checked monthly and are reported on an annual basis (1^st ^April to 31^st ^March). To maintain client anonymity, data are collected in a pseudo-anonymous form with an individual identified by their 'attributor code' comprised of their initials, date of birth, sex and their D(A)AT of residence.

The NDTMS database for the North West of England (includes the Cheshire and Merseyside, Cumbria and Lancashire and Greater Manchester geographies) was interrogated to retrospectively identify all individuals who had a triage date, and thus began a new episode of treatment, within the 2005/06 reporting period. Measures of treatment retention were established in accordance with NTA guidance [12]. Firstly, all clients under the age of 18 were removed from the dataset because the majority of young people participate in short-term interventions not designed to last for 12 weeks. The triage and discharge dates were used to determine the length of each treatment episode. Where two episodes for the same person overlapped (the treatment start date of the second episode being before the discharge date of the first episode), the individual was recorded as being continuous in the treatment system and the triage date of the first episode and discharge date of the second was used to identify the length of contact. An individual was also considered to be in continuous treatment if the start date of a second episode of treatment was within three weeks of the discharge date of the first episode of treatment, even if they dropped out prematurely from the first agency (three weeks reflects the effectiveness target that an individual should be seen within three weeks of being referred to a service [12]). An individual is defined as being retained in treatment if their total treatment episode within the system was 12 weeks or longer. An individual was deemed to have dropped out prematurely if their total treatment episode lasted for less than 12 weeks and the reason for discharge was coded as 'dropped out/left'. Individuals who were not in the treatment system for 12 weeks who had other discharge reasons (such as 'died', 'sent to prison') were excluded from the analyses. To avoid correlations in the data and adhere to the NTA's methodology for retention analysis, attributor codes were used to remove double counting of individuals within the dataset. Drop out or retention status was defined using an individuals' most recent treatment episode.

Other variables obtained from the NDTMS database were sex, age at triage assessment, ethnicity code, D(A)AT of residence, route of referral into treatment and up to three drugs. D(A)AT of residence was recoded to public health zone, and route of referral was recoded into criminal justice or otherwise. North West NDTMS data for 2003/04 and 2004/05 were used to identify whether each individual had been in contact with treatment within these two periods (used to define 'recent contact'). The variables included in the final dataset are detailed in Table 1. Deprivation was assessed using a population weighted Index of Multiple Deprivation (IMD) that was available for each D(A)AT from the North West Public Health Observatory. The IMD (2004) combines indicators collected from routine data relating to the following seven domains: income, employment, health and disability, education, skills and training, barriers to housing and services, living environment and crime [17]. A higher IMD score indicates a greater level of deprivation. Based upon their IMD scores, D(A)ATs were separated into deprivation quintiles (five D[A]ATs were allocated to quintiles one and five, and four each to quintiles two, three and four; Table 2). Each individual was assigned a deprivation score based upon their D(A)AT of residence. Data manipulation and matching was undertaken using Microsoft Access.

**Table 1:** Description of the variables included in the final dataset

**Variable**	**Description**
Attributor code (initials, date of birth, sex and D[A]AT of residence)	Used to identify an individual
Sex	Female or male
Age at triage	Categorised into 18–24, 25–34, 35–44, 45–54 and 55–74
Date of triage and discharge	Used to identify the start and end of each episode of treatment
Outcome code	Retained, dropped out or other (see results section for other outcomes)
Ethnicity code	White (white British, white Irish, white other) Mixed (white and black Caribbean, white and black African, white and Asian, other mixed) Asian/Asian British (Indian, Pakistani, Bangladeshi, other Asian) Black/Black British (Caribbean, African, other black) Other (Chinese, other)
D(A)AT of residence	Recoded to public health zone of residence Cheshire and Merseyside (Cheshire, Halton, Knowsley, Liverpool, Sefton, St Helens, Warrington, Wirral) Cumbria and Lancashire (Blackburn, Blackpool, Cumbria, Lancashire) Greater Manchester (Bolton, Bury, Manchester, Oldham, Rochdale, Salford, Stockport, Tameside, Trafford, Wigan)
Referral source	Recoded to criminal justice and non-criminal justice referral route Non-criminal justice referral route (Accident and Emergency departments, Community Care Assessment, Connexions, drug service: non-statutory, drug service: statutory, Education Services, Employment Services, General Practitioner, looked after children, Psychiatry, self, Social Services, needle exchange services) Criminal justice referral route (Arrest Referral/Drug Interventions Programme, CARAT, Drug Treatment and Testing Order, Probation, Youth Offending Team)
Recent treatment contact	No (no treatment contact in 2003/04 or 2004/05) or yes (treatment contact in 2003/04 or 2004/05)
Opiate use	No or yes (heroin, methadone, other opiates)
Stimulant use	No or yes (amphetamine, cocaine, crack cocaine)
Alcohol use	No or yes
Deprivation	Assessed using a composite score: the Index of Multiple Deprivation. The index is based on data for seven domains: income, employment, health and disability, education, skills and training, barriers to housing and services, living environment and crime. A higher score reflects a higher level of deprivation. Table 2 shows the scores for each D(A)AT area and the quintiles used for the analyses.

**Table 2:** Deprivation quintile of Drug (and Alcohol) Action Teams

**Drug (and Alcohol) Action Team**	**Index of Multiple Deprivation score^1^**	**Deprivation quintile (1 = most deprived, 5 = least deprived)**
Liverpool	49.82	1
Manchester	48.95	1
Knowsley	46.58	1
Salford	38.19	1
Halton	34.29	1
Blackpool	33.91	2
Rochdale	33.71	2
Blackburn	32.29	2
St Helens	31.95	2
Oldham	30.73	3
Wirral	30.06	3
Tameside	29.81	3
Bolton	29.41	3
Wigan	29.27	4
Sefton	26.12	4
Bury	23.53	4
Lancashire	21.80	4
Cumbria	21.57	5
Trafford	20.14	5
Warrington	19.33	5
Stockport	18.06	5
Cheshire	15.06	5

^1 ^Source: North West Public Health Observatory, Centre for Public Health, Liverpool John Moores University

### Data analysis

All variables were investigated for erroneous or missing values. Bivariate analyses were conducted on all data. Only records with complete data were included in multivariate analyses to ensure that Likelihood Ratio Tests (LRT) compared nested models. For the purpose of this analysis, the quantitative variable 'age at triage' was categorised as follows: 18–24, 25–34, 35–44, 45–54 and 55–74.

Bivariate analyses between outcome (retention or drop out) and all explanatory variables were assessed independently using odds ratios and chi-square analyses. Forward stepwise logistic regression was then used to identify factors associated with drop out following simultaneous adjustment for other variables. Variables whose significance in the bivariate analyses was P ≤ 1.0 were entered into the model and their effects tested separately. Variables were entered into the model in the following order: age, ethnicity, opiate user, alcohol user, recent treatment contact, public health zone and deprivation. Interactions between age and all other explanatory variables were tested because previous research in the UK showed drug treatment outcomes varied significantly with age [18].

The LRT was used for three analyses. 1) to test the null hypothesis that the variable under consideration was not significantly associated with drop out; 2) to compare a model with a linear trend with a model with individual effects across strata for ordinal explanatory variables (age group and deprivation quintile) and 3) to compare a model with and without an interaction term between age group and, separately, each other variable. A LRT statistic of P < 0.05 indicated significant differences between the two models being compared. For model simplicity, common odds ratios were used where a linear trend was identified and interaction terms were not included in the model if the interaction term did not significantly improve the model's fit. Additive models were not considered because odds were being modelled. Pearson's chi-square was used to evaluate the fit of the model to the data. All analyses were undertaken using STATA intercooled version 8 [19].

The Ethics Committee of the London School of Hygiene and Tropical Medicine granted ethical approval.

## Results

In 2005/06, 22330 triage assessments were recorded by the NDTMS database for the North West of England for drug users aged between 18 and 74. This figure included multiple triage assessments for the same individual when they had been seen by two different treatment services or had started a new period of treatment at the same service on multiple occasions. Following data preparation in the manner detailed above, 16626 individuals aged between 18 and 74 began a new episode of treatment in the North West of England in 2005/06. Data on treatment outcomes were unavailable for 272 (1.6%) individuals. Of the remaining 16354 people, 12230 (74.8%) were retained in treatment for 12 weeks or more and 1649 (10.1%) dropped out of treatment before 12 weeks. A further 2475 (15.1%) episodes resulted in other treatment outcomes. These other treatment episode outcomes were as follows: treatment completed in less than 12 weeks, client drug free (n = 275, 1.7%), treatment completed in less than 12 weeks, client not drug free (n = 326, 2.0%), treatment withdrawn, contract breached by client (n = 379, 2.3%), no appropriate treatment available (n = 106, 0.6%), treatment declined by client (n = 1, <0.1%), inappropriate referral (n = 26, 0.2%), client referred on (n = 555, 3.4%), client moved away from area (n = 103, 0.6%), client went to prison (n = 308, 1.9%), client died (n = 23, 0.1%), other outcome (n = 220, 1.3%), outcome not known (n = 153, 0.9%).

The characteristics of those dropping out of treatment and those retained are displayed in Table 3. Bivariate analyses showed that drop out was significantly associated with seven variables (Table 4). Firstly, those who had recently been in contact with treatment were less likely to drop out than those who had not (odds ratio 0.84, 95% confidence interval 0.76 to 0.93). Secondly, the odds of drop out were significantly higher among residents of Cumbria and Lancashire (odds ratio 1.65, 95% confidence interval 1.43 to 1.90) and Greater Manchester (odds ratio 1.90, 95% confidence interval 1.68 to 2.16) than residents of Cheshire and Merseyside. For those people who were resident in areas categorised as being in deprivation quintiles two to five, the odds of drop out were significantly greater than the odds of drop out for those living in the most deprived areas (quintile one). The relationship between age and treatment outcome showed an inverse linear trend, with the odds of drop out decreasing with increasing age. With respect to the drug profile, the odds of drop out was significantly lower for individuals who were opiate users (odds ratio 0.79, 95% confidence interval 0.71 to 0.88), and for alcohol users (odds ratio 0.74, 95% confidence interval 0.59 to 0.91). The use of alcohol was recorded for 435 (4.4%) opiate users and 574 (9.8%) stimulant users. Only 150 individuals were recorded as using all three categories of drugs, while 1654 were reported as not using any of these drugs (and were users of cannabis or benzodiazepines for example). Finally, the odds of drop out varied according to ethnicity, with Asian drug users more likely to drop out than their white counterparts (odds ratio 1.97, 95% confidence interval 1.45 to 2.67). Other variables were not significantly associated with treatment outcome.

**Table 3:** Characteristics of those dropping out of, and retained in, drug treatment (N = 13879)

**Explanatory variables**	**Retained (controls) – n (%)**	**Drop out (cases) – n (%)**
**SITUATIONAL/ORGANISATIONAL FACTORS**		

**Recent treatment contact**		
No	5476 (44.8%)	809 (49.1%)
Yes	6754 (55.2%)	840 (50.9%)
**Referral route**^1^		
Non-criminal justice	7801 (73.2%)	1084 (74.6%)
Criminal justice	2854 (26.8%)	370 (25.4%)
**Area of residence (public health zone)**		
Cheshire and Merseyside	4702 (38.4%)	424 (25.7%)
Cumbria and Lancashire	2942 (24.1%)	438 (26.6%)
Greater Manchester	4586 (37.5%)	787 (47.7%)
**Deprivation**		
1 (most)	3465 (28.3%)	364 (22.1%)
2	1800 (14.7%)	286 (17.3%)
3	2268 (18.5%)	328 (19.9%)
4	2979 (24.4%)	388 (23.5%)
5 (least)	1718 (14.0%)	283 (17.2%)

**PERSONAL CHARACTERISTICS**		

**Sex**		
Female	3359 (27.5%)	438 (26.6%)
Male	8871 (72.5%)	1211 (73.4%)
**Age group (years)**		
18–24	1902 (15.6%)	362 (22.0%)
25–34	5487 (44.9%)	780 (47.3%)
35–44	4071 (33.3%)	436 (26.4%)
45–54	654 (5.3%)	64 (3.9%)
55–74	116 (0.9%)	7 (0.4%)
**Ethnicity**^2^		
White	11343 (95.3%)	1462 (93.5%)
Mixed	142 (1.2%)	23 (1.5%)
Asian/Asian British	209 (1.8%)	53 (3.4%)
Black/Black British	130 (1.1%)	19 (1.2%)
Other	76 (0.6%)	7 (0.4%)

**DRUG USE**		

**Opiate user**		
No	3530 (28.9%)	560 (34.0%)
Yes	8700 (71.1%)	1089 (66.0%)
**Stimulant user**		
No	7087 (57.9%)	961 (58.3%)
Yes	5143 (42.1%)	688 (41.7%)
**Alcohol user**		
No	11254 (92.0%)	1550 (94.0%)
Yes	976 (8.0%)	99 (6.0%)

^1 ^N = 12109; for 354 individuals the data were missing and for a further 1416 'other' was recorded so it was not possible to identify whether or not the referring agency was a criminal justice service.

^2 ^N = 13464

**Table 4:** Factors affecting drop out from drug treatment, bivariate analyses (N = 13879)

**Explanatory variables**	**Mantel-Haenszel odds ratio (95% confidence interval)**
**Recent treatment contact**	
No	Reference
Yes	0.84*** (0.76 – 0.93)
**Referral route**^1^	
Non-criminal justice	Reference
Criminal justice	0.93 (0.82 – 1.06)
**Area of residence (public health zone)**	
Cheshire and Merseyside	Reference
Cumbria and Lancashire	1.65*** (1.43 – 1.90)
Greater Manchester	1.90*** (1.68 – 2.16)
**Deprivation**	
1 (most)	Reference
2	1.51*** (1.28 – 1.78)
3	1.38*** (1.17 – 1.61)
4	1.24** (1.07 – 1.44)
5 (least)	1.57*** (1.33 – 1.85)
**Sex**	
Female	Reference
Male	1.05 (0.93 – 1.18)
**Age group (years)**	
18–24	Reference
25–34	0.75*** (0.65 – 0.85)
35–44	0.56*** (0.48 – 0.65)
45–54	0.51*** (0.39 – 0.68)
55–74	0.32** (0.15 – 0.69)
**Ethnicity**^2^	
White	Reference
Mixed	1.26 (0.81 – 1.96)
Asian/Asian British	1.97*** (1.45 – 2.67)
Black/Black British	1.13 (0.70 – 1.84)
Other	0.71 (0.33 – 1.55)
**Opiate user**	
No	Reference
Yes	0.79*** (0.71 – 0.88)
**Stimulant user**	
No	Reference
Yes	0.99 (0.89 – 1.09)
**Alcohol user**	
No	Reference
Yes	0.74** (0.59 – 0.91)

^1 ^N = 12109 individuals; for 354 individuals the data were missing and for a further 1416 'other' was recorded so it was not possible to identify whether or not the referring agency was a criminal justice service.

^2 ^N = 13464

** P ≤ 0.01, *** P ≤ 0.001

Logistic regression analysis (Table 5) showed that drop out continued to be significantly more likely among Asian drug users compared to white drug users in multivariate analyses (adjusted odds ratio 1.52, 95% confidence interval 1.12 to 2.08). Drop out was more likely among those living in Cumbria and Lancashire (adjusted odds ratio 1.80, 95% confidence interval 1.51 to 2.15) and Greater Manchester (adjusted odds ratio 2.00, 95% confidence interval 1.74 to 2.29) than residents of Cheshire and Merseyside and among people living in the least deprived areas (adjusted odds ratio 2.92, 95% confidence interval 1.74 to 4.90). Drop out was significantly less likely among alcohol users (adjusted odds ratio 0.73, 95% confidence interval 0.59 to 0.91,). Age group was not significantly associated with drop out once deprivation quintile was included in the model.

**Table 5:** Factors affecting drop out from drug treatment, multivariate analysis (N = 13464)

**Explanatory variables**	**Adjusted odds ratio (95% confidence interval)**
**Ethnicity**	
White	Reference
Mixed	1.14 (0.73 – 1.79)
Asian/Asian British	1.52** (1.12 – 2.08)
Black/Black British	1.10 (0.67 – 1.79)
Other	0.69 (0.32 – 1.51)
**Alcohol user**	
No	Reference
Yes	0.73** (0.59 – 0.91)
**Area of residence (public health zone)**	
Cheshire and Merseyside	Reference
Cumbria and Lancashire	1.80*** (1.51 – 2.15)
Greater Manchester	2.00*** (1.74 – 2.29)
**Deprivation**	
1 (most)	Reference
2	1.41 (0.85 – 2.34)
3	1.05 (0.65 – 1.70)
4	1.06 (0.67 – 1.70)
5 (least)	2.92*** (1.74 – 4.90)
**Age group (years)**^1^	0.88 (0.76 – 1.01)
**Effect of interaction between age group and deprivation**	
1 (most)	Reference
2	0.92 (0.75 – 1.14)
3	1.01 (0.83 – 1.24)
4	0.95 (0.78 – 1.15)
5 (least)	0.66*** (0.52 – 0.83)

Other variables entered into the model: opiate use, recent treatment contact.

^1 ^The effect of age group showed a linear trend; the adjusted odds ratio represents the common odds ratio across strata.

** P ≤ 0.01, *** P ≤ 0.001

Goodness of fit: Pearson's χ^2 ^= 322.16, P = 0.009

**Figure 1:**
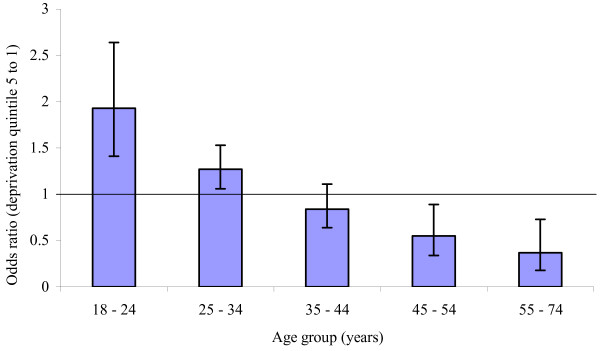
The association between deprivation and drop out from drug treatment, by age.

There was a significant interaction between age group and deprivation and the coefficients from the logistic regression model were used to explore this further. Figure 1 shows that, for those aged 18 to 24 years, the odds of drop out for deprivation quintile five (least deprived) were 1.93 (95% confidence interval 1.41 to 2.64) the odds of drop out for deprivation quintile one (most deprived). A similar, though smaller, effect was evident for the 25 to 34 age group. Conversely, for those aged 55 to 74 years, the odds of drop out for deprivation quintile five were 0.37 (95% confidence interval 0.18 to 0.73) the odds of drop out for those living in deprivation quintile one. A similar effect of smaller magnitude was observed for the 45 to 54 year age group. No interactions between age group and other explanatory variables were observed. Other explanatory variables (opiate use and recent treatment contact) were not significantly associated with treatment outcome following simultaneous adjustments for other variables. The goodness of fit test suggested that the final model was not a good fit to the data (Pearson's χ^2 ^= 322.16, P = 0.009).

## Discussion

Retention in drug treatment is considered by the NTA to be the best available measure of treatment effectiveness [4]. To encourage a reduction in drop out rates, treatment retention measures were included in local partnership treatment plans and Primary Care Trust local delivery plans for the first time in 2004/05 [12], with 75% of drug users expected to be retained in treatment for at least 12 weeks during each annual reporting period [4]. Rates of retention can be improved by better understanding factors associated with premature drop out. The aims of the present study were to assess whether the retention target was achieved in the North West of England in 2005/06 and to identify factors associated with drop out. While a number of factors associated with drop out are identified, results also highlight the complexity of factors associated with treatment outcomes.

Results show that, overall in the North West, the treatment systems achieved the Government's retention target in 2005/06, with 75% of people retained for 12 weeks or more. Contextual information provides a more valuable assessment of how services should be improved, and the analyses presented here shows that current services are failing to retain Asian drug users. Furthermore, demographic data from the 2001 census [20] shows that Asian people are underrepresented in drug treatment services while those from white, mixed, black and 'other' ethnicities are adequately represented. While people of Asian ethnicity account for 3.4% of the population of the North West [20] they accounted for just 1.9% of the drug treatment population (Table 3). This finding may reflect the lower prevalence of drug use among Asian people although a growing body of evidence indicates that drug use exists among black and minority ethnic (BME) groups and that it is increasing [21]. Qualitative research into the extent and nature of drug use among young Asians suggests that they were increasingly likely to use illicit drugs and that knowledge and use of heroin, and perhaps crack cocaine, indicated similar use to the general population of the UK [22].

An alternative explanation is that Asian drug users are not attracted by current treatment provision. A recent review of literature on drug use and service provision for BME communities in England reported a number of potential reasons for differential treatment outcomes among drug users from different ethnicities, over and above the possibility of differing drug profiles [21]. Such reasons included the ethnicity of drugs workers, communication problems for those unable to speak English and a perception among drug users that drug workers lacked awareness of their culture. No literature which evaluated outcomes of culturally-sensitive initiatives in the clinical drug treatment field in the UK could be identified by reviewers although they pointed to two USA-based studies which showed that culturally competent and culturally responsive treatment was often associated with greater treatment retention and longer treatment durations [23,24]. With respect to drug use among young Asians, healthcare professionals must be sensitive to the cultural context in which their behaviours occur and in particular the potential conflict of being affiliated to two different and at times incongruent cultural groups – British youth culture and that of their ethnic origin [22]. Further work is required to identify perceived barriers to treatment among Asian drug users and the reasons why they are more likely to drop out than their white counterparts, once contact has been initiated.

With respect to other individual characteristics, studies in the USA have reported longer durations of treatment for older drug users [8,10,25] and for male drug users [10]. Evidence from a UK-based retrospective cohort study showed no difference between men and women in rates of drop out compared to drug free discharge but that younger drug users (aged 10 to 19 years) were more likely to drop out than their older counterparts [18]. Here, bivariate analysis showed that the odds of drop out exhibited a significant inverse linear relationship with age. However, the multivariate analysis showed that age was not significantly associated with drop out once adjusted for deprivation and that a significant interaction between deprivation and age existed. The effect of deprivation was not included in the retrospective cohort study just detailed so no assessment of the relationship between age and deprivation is available.

A wealth of data exists which shows the relationship between socio-economic status and health. In the North West of England, standardised mortality rates by deprivation show that the all-age rate for those in the most deprived quintile of population is one and three quarter times the rate for the most affluent quintile of the population [26]. However, results presented here show a complex relationship between deprivation and age and their effects upon drug treatment outcomes (Figure 1). For those aged between 18 and 24 years, people living in least deprived areas were almost twice as likely to drop out of treatment than those living in the most deprived areas. A similar effect, of smaller magnitude, was observed for those aged 25 to 34 years. For those in the oldest age group, (55 to 74 years) the converse was true and those living in the most deprived areas were less likely to drop out than those living in the least deprived areas. A similar, though smaller, effect was observed for those aged 45 to 55 years.

Encouraging treatment participation among younger drug users is a well-recognised problem [14]. Younger drug users are less likely to view their drug use as problematic or view themselves as dependent upon drugs [27] and young drug users living in affluent areas may be somewhat protected from the negative consequences of drug use from their affluent families. It is not clear why older drug users living in the most deprived environmental conditions were the least likely to drop out and this finding warrants further consideration. It should be noted however, that an ecological, rather than individual, measure of deprivation was used and that affluent individuals live in deprived areas and visa versa. It is conceivable that affluent areas have a lower prevalence of problematic drug use and lower levels of service provision and that younger drug users with less motivation to change are deterred from using services to which they have to travel. Travelling more than one mile to treatment has been shown in one USA-based study to reduce the likelihood of a person completing treatment by a half after the effects of demographic differences and the type of drugs used were controlled [28] and the possible impact of travelling to services requires further consideration in the UK.

Controlling for the effect of deprivation and other explanatory factors, significant differences in the odds of drop out were observed for different areas of the North West of England with the odds greater among residents of Cumbria and Lancashire and Greater Manchester than Cheshire and Merseyside. Further work is needed to understand what is different about these regions and what factors differentially affect drop out. It is possible that there are differences between these areas in the manner in which treatment is delivered, or differences in client satisfaction with the treatment process and it is important to consider the potential influence of the quality of treatment on drop out rates rather than simply emphasising the role of client characteristics [10]. At the service rather than regional level, previous UK-based work has already demonstrated the effect the service can make on the number of clients retained successfully in treatment for six months [29]. The time people wait to access treatment will also influence retention. Here, a person was deemed to be in continuous treatment if they reappeared in treatment within three weeks of leaving the first agency they were in contact with. Individuals who dropped out of service and did not reappear until more than three weeks had elapsed were recorded as having dropped out so geographical areas whose waiting times were longer than three weeks would have a greater proportion of people reported as dropping out. Unfortunately, no assessment of the impact of waiting times on treatment outcomes can be made in this study.

The only substance significantly associated with outcomes was alcohol, with alcohol users less likely to drop out of treatment than non-alcohol users. Following adjustment for other variables, opiate use was found not be significantly associated with treatment outcomes. The only large-scale longitudinal, prospective cohort study of treatment outcomes in the UK reported that there was no long-term reduction in drinking among patients of drug treatment programmes and authors concluded that poor drinking outcomes required urgent attention [30]. That alcohol use is considered as part of the overall drug profile of people contacting drug treatment is therefore encouraging, particularly in light of the role of alcohol in drug-related deaths [31,32] and its exacerbation of chronic hepatitis C, currently the most significant infection affecting drug users who inject in the UK [33]. It is possible, however, that this finding is a feature of reporting. The NDTMS records up to three drugs for each person, with those identified as most problematic (used most frequently, associated with greatest health and criminal consequences) reported preferentially. Alcohol is generally perceived to be less problematic than illicit drugs and individuals whose drug profile does not include alcohol may represent drug users who use a larger range of illicit drugs – those with the most severe drug problems. Alcohol use may therefore act as a proxy measure of less severe drug problems. A recent investigation into the characteristics of drug users in contact with structured drug treatment reported that those whose drug profiles included alcohol as a supplementary drug were younger, were less likely to use heroin and were more likely to be referred into treatment via the criminal justice system [34].

Previous research in the North West of England showed that those referred from the criminal justice system were significantly more likely to drop out and less likely to complete treatment drug free than those referred from non-criminal justice sources [18]. Here the route of referral was not significantly associated with treatment drop out in the bivariate analysis. Most community sentences last longer than 12 weeks; the now defunct Drug Treatment and Testing Orders for example (orders that required the offender to attend regularly both court and treatment and provide a urine sample for drug testing) became available nationally in October 2000 and could last between six months and three years [35]. It is therefore conceivable that those referred from the criminal justice system were retained in treatment for 12 weeks due to fear of breaking their order but had poorer long-term outcomes because they lacked the intrinsic motivation for behaviour change. However, the identification of poorer outcomes for criminal justice referrals [18] relates to outcomes recorded between 1998 and 2001/02, while the present study was undertaken after the introduction of retention targets. If drug use is considered a chronic condition defined by relapse and re-presentation [18,36], the present finding that a high proportion of both criminal justice and non-criminal justice referrals adhere to treatment for at least 12 weeks reflects positively on current policy and practice.

While this study investigated the effects of a variety of variables, the goodness of fit test suggests that other important covariates were missing from the model. This finding hints at the complexity of factors associated with treatment outcomes and confirms suggestions that drop out and retention is an index that capture the impact of many interrelated individual and process measures [7]. In particular, it was not possible to evaluate the effect of treatment modality on treatment retention because an individual's entire treatment episode could be comprised of treatment within different modalities, yet treatment modality is likely to have considerable bearing on treatment outcomes. An examination of the covariate patterns of the goodness of fit test showed discrepancies between the observed and expected values for deprivation and further work looking at the interaction between deprivation and other explanatory variables is needed. However, the reliability of the findings presented here is enhanced by treatment outcome data being unavailable for only 272 (1.6%) individuals. Large-scale prospective cohort studies of drug treatment outcomes are costly and time consuming and the robustness of findings are often questionable due to follow-up losses occurring differentially across comparison groups. The use of well-established monitoring systems provides a cost-effective means by which treatment outcomes can be assessed [18] and while case control studies are also prone to selection bias (with controls not adequately reflecting the exposure history of cases), here controls would have been cases if they had dropped out of treatment before 12 weeks. Recall bias, an accepted weakness of case control studies, did not affect results of this study because data were collected prospectively; exposure data recorded before treatment outcomes were ascertained.

Problems associated with the use of routine data (for example, inconsistencies in the manner in which information is recorded) are somewhat mitigated in this study by using nationally agreed definitions and reporting protocols, standardised coding framework and data validation checks [12]. Furthermore, the large sample size reduces the impact of slight non-systematic variations, increases the study power and reduces the likelihood of a Type II error. However, the study relies totally on self-reported data and the accuracy of such reports cannot be ascertained. The standardisation of the drug treatment and data reporting across England mean that the results from the North West of England can be generalised to other areas although results also show that the effect of factors specific to each region would require further consideration.

## Conclusion

During the 2005/06 reporting period, 75% of clients were retained in drug treatment in the North West of England. Asian drug users were more likely to drop out of treatment than their white equivalents, and further work into why this is the case is needed in light of evidence to suggest the drug profiles of Asian drug users are similar to those of their white counterparts. Residents of Cumbria and Lancashire and Greater Manchester were more likely to drop out of treatment than people living in Cheshire and Merseyside, and work on the impact of treatment processes, treatment quality and aspects of client satisfaction would be welcomed. While overall, those living in the least deprived areas were more likely to drop out than those living in the most deprived areas, the effect of deprivation varied significantly according to the age of the individual. The effect of deprivation on treatment outcomes requires further consideration, preferably using an individual, rather than geographically aggregated measure of deprivation. These findings show that personal characteristics, process measures and situational factors all effect treatment outcomes and highlight the complexity of factors associated with treatment success. The large sample size, small proportion of missing data and the inclusion of data quality and standardisation measures increase the validity and reliability of these findings. Finally, the NTA are currently revising the retention target, which is due to change for the 2008/09 reporting cycle. To date, it is not clear how the retention target will change, or whether 12 weeks will continue to be the retention benchmark.

## Competing interests

The authors declare that they have no competing interests.

## Authors' contributions

CMB conceived of the study and the design, conducted the data manipulation and statistical analysis and participated in writing the manuscript. AMM assisted with statistical analysis and participated in writing the manuscript. AJEM carried out the data extraction, helped identify background information and helped to write the manuscript. All authors read and approved the final manuscript.

## Pre-publication history

The pre-publication history for this paper can be accessed here:


